# Comprehensive sexuality education for out-of-school young people living with HIV and young people with disabilities: findings from a formative research study in Malawi

**DOI:** 10.1080/26410397.2023.2226345

**Published:** 2023-07-21

**Authors:** Monica Patricia Malata, Effie Kondwani Chipeta, Patani Mhango, Rose Kamanga, Deus Lupenga

**Affiliations:** a Research Fellow, Centre for Reproductive Health, Kamuzu University of Health Sciences, Blantyre, Malawi.; b Senior Research Scientist, Centre for Reproductive Health, Kamuzu University of Health Sciences, Blantyre, Malawi; c Training Coordinator, Centre for Reproductive Health, Kamuzu University of Health Sciences, Blantyre, Malawi; d Programme Analyst, Adolescents and Youth, UNFPA, Lilongwe, Malawi; e Chief Youth Officer, Malawi Ministry of Youth, Sports and Culture, Lilongwe, Malawi

**Keywords:** comprehensive sexuality education, disability, living with HIV, young people, Malawi

## Abstract

This formative study was undertaken between June 2020 and April 2021 to provide evidence to inform the design and delivery of comprehensive sexuality education (CSE) in Malawi for young people living with HIV (YPLHIV) and young people with disabilities (YPWD). The study included a desk review of the situation of these two groups and a mapping of CSE programmes and delivery approaches in Malawi. The study findings show that YPWD and YPLHIV in Malawi are marginalised groups, face stigma and discrimination, and are more vulnerable to abuse, warranting CSE that addresses their needs. Yet, they are often left out of sexuality education such as school-based programmes (due to early school drop-outs) and out-of-school programmes, as well as traditional modes. Furthermore, in instances where they have access to sexuality education, there is little evidence to suggest that the sexuality education that they receive is designed to address their needs, thus raising questions about its relevance. There is need for tailored CSE that addresses the needs of these groups and that is delivered using an approach that is easily accessible to them.

## Résumé

Cette étude formative a été entreprise entre juin 2020 et avril 2021 pour recueillir des données susceptibles de guider la conception et la réalisation d’une éducation complète à la sexualité au Malawi pour des jeunes vivant avec le VIH et des jeunes handicapés. L’étude comprenait un examen préliminaire de la situation de ces deux groupes et un inventaire des programmes et des approches de prestation de l’éducation complète à la sexualité au Malawi. Les résultats de l’étude montrent que les jeunes handicapés et les jeunes vivant avec le VIH au Malawi sont des groupes marginalisés, en butte à la stigmatisation et à la discrimination, et plus vulnérables à la maltraitance, ce qui justifie une éducation complète à la sexualité qui réponde à leurs besoins. Pourtant, ils sont souvent exclus des modes d’éducation à la sexualité tels que les programmes scolaires (en raison d’un abandon précoce des études) et les programmes extrascolaires, ainsi que des modes traditionnels. De plus, lorsqu’ils ont accès à l’éducation à la sexualité, il ne semble guère établi que l’éducation à la sexualité qu’ils reçoivent soit conçue de façon à répondre à leurs besoins, ce qui soulève des questions quant à sa pertinence. Il est nécessaire de mettre en place une éducation complète à la sexualité qui réponde aux besoins de ces groupes et qui soit dispensée au moyen d’une approche qui leur soit aisément accessible.

## Resumen

Este estudio formativo fue realizado entre junio de 2020 y abril de 2021 con el fin de generar evidencia para informar el diseño y la entrega de educación integral en sexualidad en Malaui para jóvenes que viven con VIH y jóvenes con discapacidad. El estudio consiste en una revisión documental de la situación de estos dos grupos y un mapeo de los programas de educación integral en sexualidad y los enfoques de entrega en Malaui. Los hallazgos del estudio muestran que las personas jóvenes con discapacidad y jóvenes que viven con VIH en Malaui son grupos marginados, enfrentan estigma y discriminación y son más vulnerables al maltrato/abuso, por lo cual merecen recibir educación integral en sexualidad que atienda sus necesidades. Sin embargo, a menudo estas personas no son incluidas en los programas de educación en sexualidad, tales como programas escolares (debido a que abandonan sus estudios a temprana edad) y programas fuera de la escuela, ni en modos tradicionales. Además, en casos donde tienen acceso a educación en sexualidad, existe poca evidencia que indique que la educación en sexualidad que reciben está diseñada para atender sus necesidades, por lo cual se pone en duda su pertinencia. Se necesita una educación integral en sexualidad adaptada para atender las necesidades de estos grupos y que se entregue por medio de un enfoque que les sea fácil de acceder.

## Background

Young people in Malawi face multiple health challenges, most of them related to their sexual and reproductive health and rights (SRHR). Malawi faces one of the highest HIV prevalence rates, estimated at 8.9% for the general population, with one in three new HIV infections occurring among adolescents and youth aged 15–24 years.^1^ Vulnerable adolescents and young people, including young people living with HIV (YPLHIV) and young people with disabilities (YPWD) share a compounded risk to poor sexual and reproductive health outcomes, as they have limited access to services and face stigma and discrimination.^2–4^

As children grow older, it is important that they acquire knowledge, attitudes, skills and values related to the human body, intimate relationships and sexuality through comprehensive sexuality education (CSE).^5^ CSE is described in UNESCO’s *International technical guidance on sexuality education: an evidence-informed approach* as

*“a curriculum-based process of teaching and learning about the cognitive, emotional, physical and social aspects of sexuality. The aim is to empower children and young people to: realize their health, well-being and dignity; develop respectful social and sexual relationships; consider how their choices affect their own well-being and that of others; and ensure the protection of their rights throughout their lives.”*^6^

In Malawi, CSE is delivered in-school, through the Life Skills Education curriculum that is designed to empower learners and their teachers to effectively deal with the social and health challenges and pressures affecting young people, including HIV and AIDS, teenage pregnancies, and various forms of abuses, and through various out-of-school programmes.^7^

Unfortunately, most YPWD and YPLHIV do not get the opportunity to enrol or complete their education for reasons including the lack of reasonable accommodations within schools, discrimination and the lack of support from their carers, and thus miss out on the opportunity to obtain CSE in school.^8–10^ We undertook a formative study to inform the design of implementation research that aims to generate evidence on delivery of CSE for out-of-school YPLHIV and YPWD in Malawi. The implementation research in Malawi is part of the *Reaching those most left behind through CSE for out-of-school young people* initiative which is being undertaken in Malawi, Ethiopia, Ghana and Colombia, with the support of the United Nations Population Fund (UNFPA). This paper will focus on understanding the demographic and social context of YPLHIV and YPWD, the scope of out-of-school CSE, and how the different delivery channels of CSE reach YPLHIV and YPWD in Malawi.

## Methods and analysis

### Study design

This formative study was carried out between June 2020 and April 2022 to inform the design for the delivery of CSE to out-of-school YPLHIV and YPWD. We used a desk review and a mapping exercise of programmes delivering CSE in Malawi to understand the context of YPLHIV and YPWD. The desk review involved searching the databases Medline and Google Scholar, and manual searching of any material related to CSE in Malawi published between 1st January 2000 and 31st March 2022. We relied on the expertise of the UNFPA Malawi Partner Network and the Malawi Ministry of Youth to identify CSE programmes or interventions being implemented in Malawi. We also used Google searches and organisation websites to identify any material related to CSE in Malawi. The mapping aimed at identifying the content covered and focus population (see Table 1). We searched the terms (“comprehensive sex education” OR “sex education” OR “sexua*” “education” OR “sexua*”) “information” AND (“Malawi”).

**Table 1.** Mapping of CSE programmes in Malawi.

Organisation programme name	Population reached	Geographical area	Topics covered
Save the Children, Comprehensive Sexuality Education and Family Planning for Protection and Empowerment of Adolescents and Women Project (CSEFP)	In-school and out-of-school clubs. Adolescent boys and girls aged 10–24 years	Rumphi, Mzimba, Nneno, Ntchisi, Mwanza,	Sexual and reproductive health and rights; youth empowerment through village loans and savings; family planning and safer sex; values, rights, culture and sexuality; skills for health and well-being; sexuality and sexual behaviour; sexual and reproductive health; **HIV and AIDS stigma**
Village Reach, Health Center by Phone (Chipatala Cha Pa Foni)	All adolescent age groups. In-school and out-of-school	Machinga, Zomba, Balaka	STI’S; maternal and child health; family planning methods
United Nations Children’s Fund, Poverty reduction through improved quality education and basic life skills for in an out of school adolescent girls in Malawi	In- and out-of-school Adolescents; standard 5 to 8, aged 10–19 years	Mangochi, Salima, Dedza	Vocational skills; life skills for SRH; basic livelihood skills
Youth Project Excellent, Youth project excellence	Adolescent girls and women aged 13–24 years In- and out-of-school, also includes **people with disabilities**	Chiradzulu	Sexual and reproductive healthcare services; sexuality information; sexuality education; respect for bodily integrity; sexual agency and choosing their partners and consensual marriage; enjoyable and pleasurable sexual life
UNESCO, Our right, Our lives, Our future (O3) initiative	Adolescents ages 15–19	Ghana, Eswatini, Malawi, Tanzania, Zambia, and Zimbabwe	Relationships; values, rights, culture and sexuality; understanding gender-based violence and staying safe; skills for health and well-being; sexuality and sexual behaviour; sexual and reproductive health
UNFPA, Safeguard Young People Programme	Adolescents aged 15–24 years; in- and out-of-school young people	Nkhatabay, Mchinji, Dedza, Mangochi, Chiradzulu, Chikwawa	Values and rights; adolescents’ development; sexuality; gender roles and equality; planning for the future; relationships; communication; pregnancy; **STIs and HIV;** prevention and risk reduction; sexual and gender-based violence; advocacy activities
UNICEF, UN Joint Programme on Girls Education	In- and out-of-school adolescents; standard 5 to 8, aged 10–14 years; also boys and girls aged 15–19 years	Dedza, Salima, Mangochi	Values and rights: adolescents’ development; sexuality; gender roles and equality; planning for the future; relationships; communication; pregnancy; **STIs and HIV**; prevention and risk reduction; sexual and gender-based violence; advocacy activities
Youth Network and Counselling, Spotlight Initiative	Out-of-school young people	Mzimba, Nkhatabay, Nsanje, Machinga, Ntchisi, Dowa.	Values and rights; adolescents’ development; sexuality; gender roles and equality; planning for the future; relationships; communication; pregnancy; **STIs and HIV**; sexual and gender-based violence; advocacy activities
Association of Progressive Women, Securing Children’s Rights through Education and Protection	Adolescents aged 15–19 years Both boys and girls	Mwanza	Growth and development; gender; **HIV/AIDS (Post exposure prophylaxis (PEP), Positive living);** assertiveness
Farmer’s Union, Adolescent Nutrition Sensitive Agriculture- ANSA	Adolescents aged 15–19 years Both boys and girls	Mwanza	Sex and gender roles; **HIV/AIDS and STIs**; contraceptives; menstrual health management & hygiene; human development; self-esteem and assertiveness; relationships; critical thinking and decision making; stress and anxiety. Management; leadership; management of peer pressure; alcohol and drug abuse; counselling and psychosocial support.
Passion for Women and Children, She Matters Project (Menstrual Hygiene)	School-going girls/School structures		Values, rights, culture, and sexuality; understanding gender: skills for health and wellbeing; The human body and development
Population Services International, Nzatonse Project	Male and Female youth aged 10–24 years, men and women in reproductive age group 15–49	Ntchisi	Sexual and reproductive health: Pregnancy and pregnancy prevention; **HIV and AIDS stigma, care, treatment, and support; Understanding, recognising, and reducing the risk of STI’s, including HIV**
Ladder for Rural Development Organisation, Ending VAWG/HP in Malawi	Women of reproductive age and girls	Ntchisi	Relationships; values, rights, culture, and sexuality; understanding gender; violence and staying safe; skills for health and well-being; the human body and development; sexuality and sexual behaviour; sexual and reproductive health; **HIV and AIDS stigma**
Evangelical Association of malawi-(EAM), Nzatonse SRHR (Sexual Reproductive Health and Rights)	Youths aged 10–25 years, men & women of reproductive age	Ntchisi	Relationships; values, rights, culture, and sexuality; understanding gender; violence and staying safe; skills for health and well-being; the human body and development; sexuality and sexual behaviour; sexual and reproductive health; **HIV and AIDS stigma**
Plan International Malawi, Adolescent Girls and Young Women: AIDS Free Girls and Education (AGE)	In-school and out-of-school girls and boys aged 10–24 years	Lilongwe peri-urban	Building your dreams; menstruation; pregnancy prevention; communication; social power and gender; **HIV and Living with HIV;** goal setting
Blantyre Synod, N’zatonse Project	Boys and girls aged 18–30 years	Blantyre, Thyolo	Sexual and reproductive health rights; human sexuality; adolescence; puberty; the reproductive system; menstruation; abstinence and postponing sex; pregnancy; planning our future; abortion; STIs, **HIV & AIDS;** culture; gender; socialisation; dating and rape; alcohol and drug abuse and decision making
Pakachere Institute, Reproductive health advocacy partnership	Boys and girls aged 15–24 years	Blantyre, Dedza	Values and rights; adolescence; gender roles and equality; planning for the future; relationships; communication; **STIs and HIV** prevention and risk reduction; sexual and gender-based violence
CAMFED, CAMFED Association	Adolescent girls both in school and out-of-school	Zomba, Machinga	Getting to know each other; adolescence and sexual and reproductive health; gender and power relations; **STIs and HIV** in adolescents; (**HIV Knowledge, HIV Testing, Positive Living).**
	Adolescent girls and young women aged 10–19	Zomba, Machinga	Building your dreams; team building; safe environment; your body, your health; communicating with others; social, power and gender; feeling power and powerlessness; **HIV and You;** your future
Mponela AIDS Information and Counselling Centre, Education for Life	In-school adolescents (aged 10–24 years) and out of school (aged 14–25 years) **Youth living with HIV** as another special focus group (aged 10–25 years)	Dowa	Peer leadership; knowing and accepting present realities; life skills for **AIDS-free generation**
Family Planning Association of Malawi, Access	In- and out-of-school youths aged 10–24 years	Karonga, Dedza, Kasungu, Ntcheu, Lilongwe, Dowa	**STIs and HIV/AIDS**; condom use and promotion; family planning; cervical cancer; growing up and body changes; sexual orientation
Feed the Children, Tiwalele 2	Adolescents aged 15–19 years (Lactating adolescent girls)	Karonga, Dowa, Mchinji, Salima, Blantyre City, Blantyre District, Lilongwe City, Lilongwe District, Mulanje, Nkhotakota and Thyolo	Discovering my strengths; nutrition; Understanding Our Bodies; understanding your body; sexuality; abstinence; **STIs and HIV and AIDS**, gender dynamics and gender-based violence; goal setting
Help a Child, YACSMART project: Youth Active in Climate Smart Agriculture	Youths aged 18–35 years	Mzimba, NK, Rumphi,	Growing up and body changes; family planning; **STIs and HIV/ AIDS**; relationships; gender; gender-based violence; gender roles; sexual orientation; sexual satisfaction; condom use and promotion; cervical cancer etc.
Ujama Pamodzi, JPGE	Adolescent girls	Salima	Problem solving; decision making; effective communication; peaceful conflict resolution; interpersonal relationships; good health good hygiene.
More Than Brides Alliance (Save The Children, YONECO GENET, Marriage no Child Play) (More Than Brides)	Adolescent girls	Mchinji, Nkhatabay, Mangochi,	Peer education; life skills; adolescence and puberty; family planning methods; sexual abuse; **HIV testing**
International Organisation for Migration, SRHR-HIV Knows No Border project	Migrants, adolescents and young people and sex workers	Mchinji, Mwanza-Neno	**SRHR-HIV and migration services;** SRHR services for migrants and sex workers; referral networks
Kachila Youth Initiative, TUBAPOKE (Let`s Protect Girls and Boys from child marriages and other sexual Reproductive Health Rights)	In- and out-of-school youths aged 10–24 years	Karonga	Peer education; sexuality & knowledge; gender & human rights
FOCUS, Reaching Mother & Child for Health	In- and out-of-school youth and adolescent girls (aged 10–24 years)	Karonga	Gender & social inclusion; human rights; family planning
FOCUS, Tithetse Nkhanza	Women & girls	Karonga	Gender-based violence; teenage pregnancies and child marriage; laws around child marriages
Malawi Girl Guide Association, Safeguards Young People Program (SYP)	Both in- and out-of-school, aged 10–14, 5–19, and 20–24 years	Dedza, Mangochi, Chiradzulu, Chikwawa, Mchinji	Values and rights; adolescent growth and development; sexuality; gender roles and equality; planning for the future; relationships; effective communication; pregnancy; **STIs and HIV**, sexual and gender-based violence; advocacy
Malawi Girl Guide Association, Joint Program on Girls Education	Both in- and out-of-school, aged 10–14, 5–19, and 20–25 years	Dedza, Salima, Mangochi	Values and rights; adolescent growth and development; sexuality; gender roles and equality; planning for the future; relationships; effective communication; pregnancy; **STIs and HIV**; sexual and gender-based violence; advocacy
Malawi Girl Guide Association, Reaching Those Most Left Behind With CSE	Both in- and out-of-school, aged 10–14, 5–19, and 20–26years	Dedza, Mangochi, Chiradzulu, Chikwawa, Mchinji	Values and rights; adolescent growth and development; sexuality; gender roles and equality; planning for the future; relationships; effective communication; pregnancy; **STIs and HIV**; sexual and gender-based violence; advocacy
Malawi Girl Guide Association, Comprehensive Action For Adolescent Girls and Young Women	Both in- and out-of-school, aged 10–14, 5–19, and 20–27years	Mulanje	Values and rights; adolescent growth and development; sexuality; gender roles and equality; planning for the future; relationships; effective communication; pregnancy; **STIs and HIV**; sexual and gender-based violence; advocacy
Malawi Girl Guide Association, Girl Guiding for sexual and reproductive health and rights	Both in- and out-of-school, aged 10–14, 5–19, and 20–28years	Lilongwe	Values and rights; adolescent growth and development; sexuality; gender roles and equality; planning for the future; relationships; effective communication; pregnancy; **STIs and HIV**; sexual and gender-based violence; advocacy
Youth Initiative for Change and Development, Adolescents sexual Reproductive Health Rights	Adolescents of ages 1024.	Lilongwe, Mchinji, DOWA, Ntcheu, Nneno, Mwanza, Mulanje	All topics in CSE manual
CRIDOC, CSE	Adolescents aged 10–24 years	Lilongwe urban and rural, Nkhotakota	Menstrual health; psychosocial health; HIV prevention; linkages with health facilities
DREAMS (Determined, Resilient, Empowered, AIDS-free, Mentored and Safe) Project, SSDI – Communication /Johns Hopkins Centre for Communication Programs	AGYW aged 10–19 years	Zomba, Mchinga, Mangochi	Building your dreams; understanding your body; pregnancy; communicating with others; decision making; gender dynamics; sexual and reproductive health rights; **HIV Knowledge and Testing, HIV Stigma and Support and Living with HIV**; goal setting
Bantwana World Initiative, Protect Our Youth	Adolescents aged 13–18 years, their caregivers, and school authorities	Chiradzulu, Kasungu, Mzimba, Blantyre	*My dreams, My choice guide*: goal setting; safe environment; knowledge of one’s body; menstruation; pregnancy; condoms; communication; girls’ vulnerability to **HIV/AIDS**; gender or sex; gender equity as protection from **HIV;** gender dynamics and gender-based violence
International Planned Parenthood Federation, Get Up Speak Out	Out-of-school youth and adolescents (aged 10–24 years)	Mangochi and Chikwawa	Sexual health rights; CSE messaging for **Young People Living with HIV/AIDS**, **Young People with** d**isabilities** etc. access to SRH services; youth advocacy

### Studies inclusion and exclusion criteria

We looked at information on the demographic characteristics and context of YPLHIV and YPWD and at CSE programmes reaching (or not reaching) them. We excluded information on programmes with a primary focus on education but with no clear component of sexuality education or SRH information directed towards young people in Malawi.

### Data synthesis and analysis

We used a narrative analysis to explore the situation and context of YPLHIV and YPWD in relation to the SRHR problems that they face. We synthesised the key themes and messaging for different CSE programmes in comparison with the needs of YPWD and YPLHIV. We further explored how existing programmes and methods of delivery include or fail to include these young people. We then summarised findings to identify the gaps that exist in the current CSE for YPWD and YPLHIV in Malawi.

## Results

### The situation of YPWD in Malawi

In Malawi, it is estimated that 10.4% of the population over 5 years old have at least one type of disability.^11^ The most common types of impairment are limited sight, mobility and hearing. Evidence shows that rural and poor residents are more likely to have impairments leading to disabilities than their urban and wealthy counterparts. Furthermore, some impairments are treatable or can be corrected and yet persist due to inadequate access to health care^12^ and assistive aids (such as corrective glasses). Thus, there is a strong linkage between poverty and disability in Malawi.

YPWD represent a vulnerable group due to stigma and discrimination which hinder them from social, economic, political and physical participation. Most of these young people are poor and rural residents and have limited access to health services for several reasons. First, they lack access to services due to long distances since most of them are from remote rural areas; this is exacerbated by poverty which limits their ability to finance their transportation.^4^ Second, in instances where they are able to get to health facilities, they also face systematic barriers within health facilities, such as the lack of accommodative resources like use of sign language for those with hearing impairment,^13^ braille resources, and trained service providers for young people with special needs. Furthermore, people with disabilities are culturally presumed to be asexual and are often not targeted with SRHR messages. They also face abuse from service providers and other service users at point of service,^13,14^ thus further pushing them away from services.

The Government of Malawi has made great strides to ensure that YPWD have access to formal education, yet 35% of YPWD have never attended any education compared to 18% of the general population.^15^ More YPWD drop out of school earlier due to limited resources to accommodate their needs^12,16,17^ such as skilled teachers, braille facilities for the blind, different font materials for young people with albinism and hearing aids for young people with hearing impairments.^18^ YPWD enrolled in mainstream education also face discrimination and isolation from their peers, further pushing them to drop out.^16,18^ They may also drop out earlier as a result of frequent illness or chronic pain associated with their impairment.^19^ In some instances, families may also not enrol their children due to the belief that their child cannot be educated without the resolution of their impairment.^12^ Since CSE forms part of the curriculum, most YPWD thus miss out on the opportunity to attain school-based CSE.

### The situation of YPLHIV in Malawi

HIV remains a big problem in Malawi. Adolescent girls and young women aged 15 to 24 years have a disproportionate burden of HIV, and are four times more likely to get infected than their male counterparts.^1^ In addition, young people living in the urban areas or Southern region have a worse burden of infection than their rural or Central or Northern region counterparts.

Despite various campaigns to end HIV stigma within communities, YPLHIV still face a lot of stigma and discrimination associated with their own or their guardian’s HIV-positive status.^10,20,21^ HIV stigma is based on misconceptions which devalue the social status of people living with HIV within communities, such that other members of the community may not wish to be associated with them. For instance, HIV infection is often attributed to promiscuity, which is culturally frowned upon. Furthermore, incorrect information about the transmission mechanisms of HIV pushes them into isolation, as people may fear getting infected by sharing items such as utensils and clothing with them.^20,22,23^ Thus, YPLHIV face stigma by being stereotyped, excluded, or discriminated against due to their HIV status.

The stigma and discrimination that YPLHIV face also has negative consequences on their mental wellbeing.^21,24^ While mental health challenges are common amongst all types of young people, YPLHIV have an increased risk of poor mental health^21,25,26^ that manifests through low self-esteem, anxiety, isolation, depression and suicide ideation, which are exacerbated by the discrimination and isolation which they face. As YPLHIV often have to hide their HIV-positive status,^10,27^ this creates anxiety as they explore intimate sexual relationships as well as friendships.

A proportion of YPLHIV were infected vertically through transmission from mother to child,^28^ increasing their chances of being orphaned.^28^ This. in turn. increases their vulnerability as they are placed in foster care, or left without any guardian,^29^ and thus exposed to poverty.^30^ As they often have to provide for themselves, YPLHIV have a higher likelihood of being exploited through involvement in child labour and transactional sex for girls.^31,32^ Their involvement in transactional sex further subjects them to risky sexual behaviours as their power within the relationships is often compromised.^32^

YPLHIV have access to education through schools; however, their completion rates are lower than the general population. Evidence suggests that YPLHIV drop out earlier for reasons that include discrimination from their peers while in school; frequent absenteeism due to AIDS-related illness; and involvement in child labour to provide for themselves.^10^

In recognition of the discrimination and unique challenges that YPLHIV face, the Teen Club Programme was introduced to provide peer support. Established in 2002, teen clubs are exclusive monthly clinics for adolescents living with HIV. They provide both clinical services and psychosocial support. Through teen clubs, YPLHIV might share their experiences in various aspects of life and encourage each other.^33^ Many facilities in Malawi have teen clubs that enrol children and adolescents living with HIV.^34^ Through teen clubs, YPLHIV interact with their peers, address their sexual and reproductive needs and also access health services. Despite this, there are many social, economic and contextual factors that are not adequately or comprehensively addressed to respond to their needs as YPLHIV.^20^ Furthermore, as they get older, they are required to transition to adult clinics, regardless of their level of preparedness to face the adult world.

### Delivery approaches for CSE in Malawi

The key delivery approaches for CSE in Malawi include a school-based approach through the life skills curriculum designed for primary school and secondary school learners; a community-based approach offered through traditional or religious channels and civil society/non-governmental organisations (NGOs) through various peer groups or teen clubs.

#### In-school sexuality education

The Government of Malawi introduced life skills for secondary and primary school learners as a response to the growing HIV and AIDS epidemic in the country since around the late 1990s.^7,35^ The curriculum combined lessons to help learners learn about their sexuality and explore life skills that would enable them to live a healthy and meaningful life. The life skills syllabuses were designed for learners from early years of primary schools all the way through to secondary school. Content is incremental as one progresses with schooling, and is also age-sensitive, designed under the assumption that younger scholars are in lower classes.^36^ The curriculum centres around the core modules on health promotion (including information on prevalent diseases such as malaria, sexually transmitted infections, HIV and AIDS); social development (relationships and gender equality); moral development (culture and religions); personal development (self-esteem, decision making and skills development) and physical development.

#### Community-based sexuality education

In Malawi, initiation ceremonies are a common practice with participation estimated at 43% for girls and 35% for boys, with more rural adolescents than urban adolescents.^37^ Initiation ceremonies are a common space in Malawi for the delivery of sexuality education, including responsible sexual and reproductive behaviour. Ceremonies take various forms; they can either be delivered by churches or cultural custodians, although the messages shared across are quite similar, but with varying levels of detail.^38^ Topics covered generally include personal hygiene, respect, sexually transmitted infections and HIV and pregnancy implications from menarche. Traditionally, elderly women are invited to talk to a girl as soon as she starts her first menstruation.^37,38^ In some instances, all girls who started their first menstruation during that year are brought together and counselled by women through initiation ceremonies where messages are put across through songs and traditional dances.^38^ We found no evidence to indicate inclusion or exclusion of YPWD; however YPLHIV may be included and encouraged to live positively.^39^

#### Civil society/NGOs sexuality education

CSE in Malawi is also delivered by NGOs. These organisations take different approaches, usually through teen clubs or peer groups. Different NGOs target different groups: for example, girls only, out-of-school youth and in-school-youth. For example, UNICEF programmes have addressed school-age children through school and out-of-school clubs and their activities have included exchange visits between clubs and the peer education and youth-led awareness campaign.^40^

### CSE mapping of programmes

Our mapping exercise shows that there are a number of CSE programmes being implemented within the country by both local and international organisations (see Table 1). The programmes reach adolescents, ages 10 years old to 19 years old, and young adults, ages 20 to 25 years old. Programmes vary, with some reaching in-school youth, others out-of-school of youth and most programmes reaching both. Most of the programmes reach young people from the general population, and only a few reach marginalised youth such as YPLHIV and YPWD. In the sampled programmes, only two indicated that they are working with YPWD, and one indicated CSE messaging that was specific for YPWD. CSE is also often delivered through teen clubs, and enrolment into these clubs is often through existing community youth networks. Thus marginalised teens who do not have pre-existing networks are often left out. Unfortunately, since YPWD and YPLHIV face discrimination and stigma, they do not often have networks outside their peers with similar characteristics, and are thus excluded from general community programmes.

The topics covered through the CSE programmes vary. Most cover topics on HIV and STIs, sexuality and sexual relationships, menstruation, pregnancy prevention and condom use, building self-esteem and goal setting, and gender-based violence and power dynamics. A few, programmes also include topics on advocacy, abortion, pleasurable sex, nutrition and vocational skills. Despite covering topics on HIV, most of the content is around prevention, such as condom use, abstinence and HIV testing, but only a few programmes cover content on living with HIV. Thus, while HIV is a popular topic, only a few programmes cover content that addresses the specific needs that YPLHIV face.

The mapping shows that most projects operate in a few districts at a time, with the number of target districts ranging from one to six districts out of the 28 districts in Malawi. This means that CSE is only delivered to a fragment of the population.

### Gaps in CSE delivery in Malawi

The gaps and challenges of current CSE programming to reach and address the needs of YPWD and YPLHIV fell under three broad categories: lack of consistency in CSE content; lack of resources and fragmentation of programming; and lack of inclusiveness of marginalised groups.

#### Lack of consistency in CSE content

CSE remains a controversial topic in Malawi, and has suffered resistance from both cultural and religious groups. As a result, the information that is delivered varies from programme to programme, with some providing less information than others.^38,39,41^ Furthermore, the information provided might also be contradictory from one programme to another. Some promote sexuality ideals such as abstinence-only and being faithful. Others may provide more information on sexuality issues that young people may face, such as acknowledging that sexual activity exists among children, as sexual beings, and including abortion and sexual orientation. This lack of consistency is across all delivery methods. Teachers may skip certain topics in a life skills curriculum to align with their personal beliefs,^36,42^ and similarly, information offered through initiation ceremonies also varies. Thus, the lack of consistency in content may result in sexuality education that is not comprehensive and sufficient to respond to the needs of YPWD and YPLHIV as sexual beings.

#### Lack of resources and fragmentation of programming

Delivery of sexuality education requires commitment and investment in resources. Unfortunately, resources for CSE have remained a challenge, resulting in fragmentation of programming. For instance, the majority of NGOs work in limited geographical areas and only reach a limited number of young people at a time. Evidence also shows a lack of appropriate teaching and learning methodologies, the need to train teachers and to develop additional materials for use in all classes.^7,43^

#### Lack of inclusiveness of marginalised groups

CSE in Malawi has followed traditional delivery approaches and very few programmes have provided CSE that is accessible to YPWD. Schools in Malawi generally lack accommodative resources such as braille facilities and sign language experts.^8,18^ Similarly, very few programmes have invested in accommodative material and there is likewise a lack of literature to suggest the inclusion of YPWD in initiation ceremonies. Thus, there is need for development of training materials specifically for marginalised groups, and investments in facilitators to support delivery of CSE that ensures no groups of young people are left behind. Further limitations to inclusion are embedded in the core design of the school syllabus, that is, more information is given in higher classes. Unfortunately, most young people drop out before acquiring any meaningful skills; 2% of children drop out after one year of schooling, 35% complete primary education (eight years of schooling) and 18% complete secondary education (12 years of schooling).^15^ Furthermore, YPWD and YPLHIV^10,15^ have an increased risk of school drop-out, thereby making it unlikely that the majority of them will have obtained CSE through education.

## Discussion

Our study set out to provide a situation analysis of YPLHIV and YPWD, map CSE programmes and explore how CSE delivery channels reach the target populations. CSE in Malawi is delivered through three main channels: in school through the life skills education curriculum; through traditional or religious initiation rites; and through civil society/NGO programmes. We mapped a range of CSE programmes with varying levels of content covered, target populations and implementation districts. Most programmes cover content on HIV and STIs, sexuality and sexual relationships, menstruation, pregnancy prevention and condom use, building self-esteem and goal setting, and gender-based violence and power dynamics. However, there are limited resources for YPWD.

Despite most programmes covering content on HIV knowledge and prevention, very few programmes are designed for YPLHIV. HIV content covered is often on condom use, HIV prevention and testing,^39^ but there is little guidance on how these young people can navigate life as individuals as well as sexual beings living with HIV. As YPLHIV, they have unique needs and also often face discrimination and stigma associated with their HIV status.^3,10,44^ The discrimination due to their HIV status affects their desire to associate with others, thus forcing them into isolation.^45^ This, in return, has an impact on their educational attainment^10^ and antiretroviral therapy retention,^3^ among other things. Thus, YPLHIV require specially curated spaces where they can freely interact with their peers without judgement while receiving skills to guide them in navigating various aspects of their lives. A study targeting adolescents living with HIV in South Africa reported that young people both found comfort in teen clubs as spaces for learning about their sexuality, and preferred accessing treatment from adolescent clinics.^3^ These findings are also echoed in other studies.^33^

While HIV topics are covered in the various programmes, there is a complete lack of content specifically designed for YPWD. Similar to their peers living with HIV, YPWD also face discrimination and isolation associated with their disability. Even in instances where they are not isolated, they require accommodations in order for them to fully access various services. Unfortunately, most public services, including health and education, do not provide these accommodative resources, thus pushing these young people into social and economic isolation. Furthermore, YPWD are vulnerable to abuse and exploitation due to their compromised physical or mental abilities. They often need to rely on other people for support, which subjects them to further vulnerability, both in the home and in the community. Despite this increased vulnerability, YPWD are culturally often assumed to be asexual, and thereby not in need of any sexuality education.^46^ Yet YPWD face an increased vulnerability to abuse. Thus, they too require safe spaces, where they are recognised as sexual beings and can learn and interact with their peers.

The mapping of programmes shows that there are a number of programmes offering sexuality education. These programmes target various groups of young people and cover various topics. They are beneficial in that they offer sexuality education for out-of-school youth, who may otherwise not have been able to gain sexuality education through schooling. Unfortunately, the programmes are also subjected to funding ceilings which affect their scope and reach, evidently with most programmes only being implemented in a few districts. As such, most of these programmes are often fragmented, reaching very few young people. Most importantly, already marginalised groups such as YPWD are also excluded from these programmes as specialised facilitators and increased resources are required in order to reach them. Beyond content, focus also has to be placed on duration of the programme, as studies show better behavioural changes for programmes with longer duration and intensity of CSE.^47^ Even for programmes reaching vulnerable groups, there is no system of monitoring of activities, and a lack of harmonisation and consistency in the content that is delivered,^42^ thus providing sexuality education that may not be comprehensive.

The current study highlights gaps in consistency in CSE content, lack of resources resulting in fragmentation of programming and the lack of inclusiveness of marginalised groups. This suggests the need for central government commitment to advancing CSE in Malawi. There is a need for policies, guidelines and standards on the design and delivery of all CSE programmes in Malawi. These guidelines would provide minimum standards for CSE programming, thereby ensuring consistency in the content as well as methods of delivery. Furthermore, central level coordination of CSE would ensure equity in the geographical allocation of CSE programmes thus ensuring that CSE reaches all, while also ensuring that marginalised groups are not left out in Malawi. Therefore, there is a need for consistent and adequate CSE to be delivered both in-school and out-of-school to ensure that no young people are left behind. Deliberate efforts must be made to target young people who are unable to access CSE through traditional channels such as schools, or young people with specific needs, including YPWD and YPLHIV, and provide them with sexuality education that is comprehensive and also adapted to their unique needs.

Our study presents only a snapshot of the situation of YPWD and YPLHIV in Malawi. We acknowledge the lack of primary research in our methods to capture the views and lived experience of the focal population and of other key informants. There is also limited literature that captures the SRHR of YPWD and YPLHIV and few studies have been done on CSE in Malawi. As a result, findings from this study are drawn from very few studies as we did not conduct a full systematic review of the literature. Furthermore, due to the limited literature, the narrative analysis techniques we employed were unsystematic,^48^ such that the findings were drawn specifically to provide a formative picture for the design of a CSE delivery intervention. Nonetheless, our study provides valuable insights into the current situation of YPWD and YLHIV and it highlights the significance of new research as well as refocused programming of CSE to meet their needs.

## Conclusions

The study describes the challenges that YPLHIV and YPWD face: stigma, discrimination and isolation, as well as being vulnerable to abuse, all of which highlight their need for CSE. The study also highlights the different programmes that are being implemented to provide CSE to young people and the various modes through which CSE is delivered. These CSE programmes are often fragmented, implemented in a few districts at a time and do not target all youth, thus leaving already marginalised populations behind. In instances where YPWD and YPLHIV may have access to CSE, they do not always receive CSE content that is appropriate for their needs. Thus, the study findings highlight the need for CSE programming that is designed to respond to the needs of YPWD and YPLHIV while also recognising the discrimination and isolation that YPWD and YPLHIV face.

Furthermore, to make CSE more inclusive, there is need for continued community engagement to make it more acceptable by changing conservative attitudes and values that hamper adolescents’ SRHR. Furthermore, facilitators of CSE need to be fully trained to meet the needs of young people while delivering sexuality education that is comprehensive and accessible to all.
